# Investigation of microbial fuel cell performance based on the nickel thin film modified electrodes

**DOI:** 10.1038/s41598-023-48290-3

**Published:** 2023-11-25

**Authors:** Fatemeh Mahmoodzadeh, Nahid Navidjouy, Saber Alizadeh, Mostafa Rahimnejad

**Affiliations:** 1 Department of Environmental Health Engineering, Urmia University of Medical Sciences, Urmia, Iran; 2 Faculty of Chemistry, Bu-Ali Sina University, Hamedan, 65174-38683 Iran; 3 Department of Chemical Engineering, Biofuel and Renewable Energy Research Center, Babol Noshirvani University of Technology, Babol, Iran

**Keywords:** Biotechnology, Environmental sciences

## Abstract

Microbial fuel cells (MFCs) are a self-sustaining and environmentally friendly system for the simultaneous was tewater treatment and bioelectricity generation. The type and material of the electrode are critical factors that can influence the efficiency of this treatment process. In this study, graphite plates and carbon felt were modified through the electrodeposition of nickel followed by the formation of a biofilm, resulting in conductive bio-anode thin film electrodes with enhanced power generation capacity. The structural and morphological properties of the electrode surfaces were characterized using X-ray diffraction, energy-dispersive X-ray spectroscopy, elemental mapping, and field-emission scanning electron microscopy techniques. Maximum voltage, current density, and power generation were investigated using a dual-chamber MFC equipped with a Nafion 117 membrane and bio-nickel-doped carbon felt (bio-Ni@CF) and bio-nickel-doped graphite plate (bio-Ni@GP) electrodes under constant temperature conditions. The polarization and power curves obtained using different anode electrodes revealed that the maximum voltage, power and current density achieved with the bio-Ni@CF electrode were 468.0 mV, 130.72 mW/m^2^ and 760.0 mA/m^2^ respectively. Moreover, the modified electrodes demonstrated appropriate stability and resistance during successful runs. These results suggest that nickel-doped carbon-based electrodes can serve as suitable and stable supported catalysts and conductors for improving efficiency and increasing power generation in MFCs.

## Introduction

Microbial fuel cells (MFCs) represent a novel and eco-friendly technology for the simultaneous treatment of wastewater and generation of bioelectricity^1,2^. The configuration of MFCs can be single-chambered or dual-chambered designs, depending on the objective of the study, dual-chambered mfc is frequently employed^3^. In Dual-Chamber MFCs (DC-MFC), the cathode anode are completely separated by a Proton Exchange Membrane (PEM), facilitating the maintenance of distinct conditions within each chamber. Moreover, the ability to integrate MFCs with other treatment methods allows for the cathode chamber to be utilized for the removal of various organic and pharmaceutical pollutants^4,5^. In the anaerobic anodic chamber, electrogenic microbes serve as active biocatalysts for the oxidation of organic materials, producing protons, electrons, and carbon dioxide as final products^6^ (Eq. 1). Electrons and protons are transferred from the anodic chamber to the cathodic chamber via the external circuit and membrane, generating an electric current through electrochemical reactions with electron acceptors^6,7^ (Eq. 2).

1 $$ {\text{C}}_{{6}} {\text{H}}_{{{12}}} {\text{O}}_{{6}} + {\text{6H}}_{{2}} {\text{O}} \to {\text{6CO}}_{{2}} + {\text{24H}}^{ + } + {\text{24e}}^{ - } $$

2 $$ {\text{H}}^{ + } + {\text{24e}}^{ - } + {\text{6O}}_{{2}} \to {\text{12H}}_{{2}} {\text{O}} $$

The generation of bioelectricity in MFCs is subject to variation under disparate conditions^8^. For example, the application of nanomaterials for the modification of anode and cathode electrode surfaces, the utilization of diverse substrates such as acetate and glucose at varying concentrations, the configuration of the cell, the type of ion exchange membrane employed, temperature, anolyte pH, internal and external cell resistance, as well as electrode distance can all exert significant influence on the electricity generation process^8^. Recent investigations have concentrated on enhancing output power and optimizing performance with in MFC systems. However, poor power generation and high cost are drawbacks that impede the widespread commercialization and practical application of MFCs^8^. To address these issues, researchers are focusing on the modification and construction of new materials for anodes and cathodes as an effective approach to improving MFC performance^9,10^. Ideal electrode materials should possess properties such as high electronic conductivity, high surface area, environmental friendliness, suitable chemical and mechanical stability, and economic affordability^10^. Carbon fibers, carbon cloth, carbon paper, carbon brushes, carbon felt, graphite, and graphene are some of the materials that have been utilized as electrodes in MFCs^11^. It is noteworthy that altering the physical and chemical characteristics of bare electrodes can increase MFC efficiency by improving electron transfer and microbial attachment^12^. In recent years, doping and modification of bare electrodes with various nanoparticles such as gold^13^ manganese^14^, platinum^15^, and its alloys^16^ have been commonly used as supported catalysts to increase current density and improve electron transfer in MFCs. However, their high cost and limited availability present obstacles to large-scale applications^17^. In recent investigations, nickel nanoparticles have been scrutinized as a coating for cathode Ni/C^18^, Ti/Ni^19^, rGO/Ni^20^, Fe/Ni^21^ and other doping methods for the anode electrode such as Ni@Fe_2_O_3_/MXene-cf^22^, owing to their cost- effectiveness, high conductivity, low resistance, and high biocompatibility. As a result, scientists have focused on the use of inexpensive and readily available catalysts with simple and eco-friendly modification techniques^23,24^. Anodic and cathodic electrodeposition^25–29^, paired electrodeposition^30^, electrochemically assisted self-assembly^31^, electro-grafting^32^ and electro-polymerization^33–35^ are green, versatile, and eco-friendly procedures for the fabrication and modification of electrodes with different electroactive thin films for targeted applications. Electroplating is a general procedure for preparing metallic thin layer films on inert substrates through the cathodic electrodeposition of dissolved or anodically released metal cations under a direct electric current density^36–38^. Black nickel and hard nickel are two different types of nickel electrodeposition. In this study, the hard nickel method was chosen due to its tensile strength and increased resistance to abrasion and corrosion^36–38^. The aim of this study was to use electroplating or cathodic electrodeposition as a simple, one-step, green, and in-situ method for modifying carbon felt and graphite plate with a nickel thin film, resulting in a low-cost, highly conductive, and highly resistant electrode. Subsequently, the nickel-doped electrodes were post-modified with microorganisms to create novel and efficient bio-anode materials through the growth of biofilms. To the best of our knowledge, no previous studies have reported on the cathodic electrodeposition of bio-nickel doped carbon felt (bio-Ni@CF) or bio-nickel doped graphite plate (bio-Ni@GP) electrodes for use as bio-anodes in an MFC system. In this comprehensive and comparative study, various parameters such as maximum voltage, current density, power generation, and electrode stability were investigated using a dual-chamber microbial fuel cell equipped with bio-Ni@CF and bio-Ni@GP electrodes under constant temperature conditions for bioelectricity generation.

## Experimental section

### Chemical and materials

Potassium dihydrogen phosphate (KH_2_PO_4_), dipotassium hydrogen phosphate (K_2_HPO_4_), ammonium chloride (NH_4_Cl), glucose (C_6_H_12_O_6_, H_2_O), calcium chloride (CaCl_2_), magnesium sulfate (MgSO_4_), potassium chloride (KCl), sodium chloride (NaCl), sulfuric acid (H_2_SO_4_ 95.0–97.0% purity), hydrochloric acid (HCl 37.0%), sodium hydroxide (NaOH), Nafion 117 (Sigma-Aldrich), peptone, yeast, anhydrous sodium sulfate (Na_2_SO_4_), boric acid (H_3_BO_3_), were purchased from Merck Company and used without further purification. All aqueous solutions were prepared daily using distilled water at room temperature. All chemicals used were of analytical grade.

### Preparation of modified electrodes

**Figure 1:**
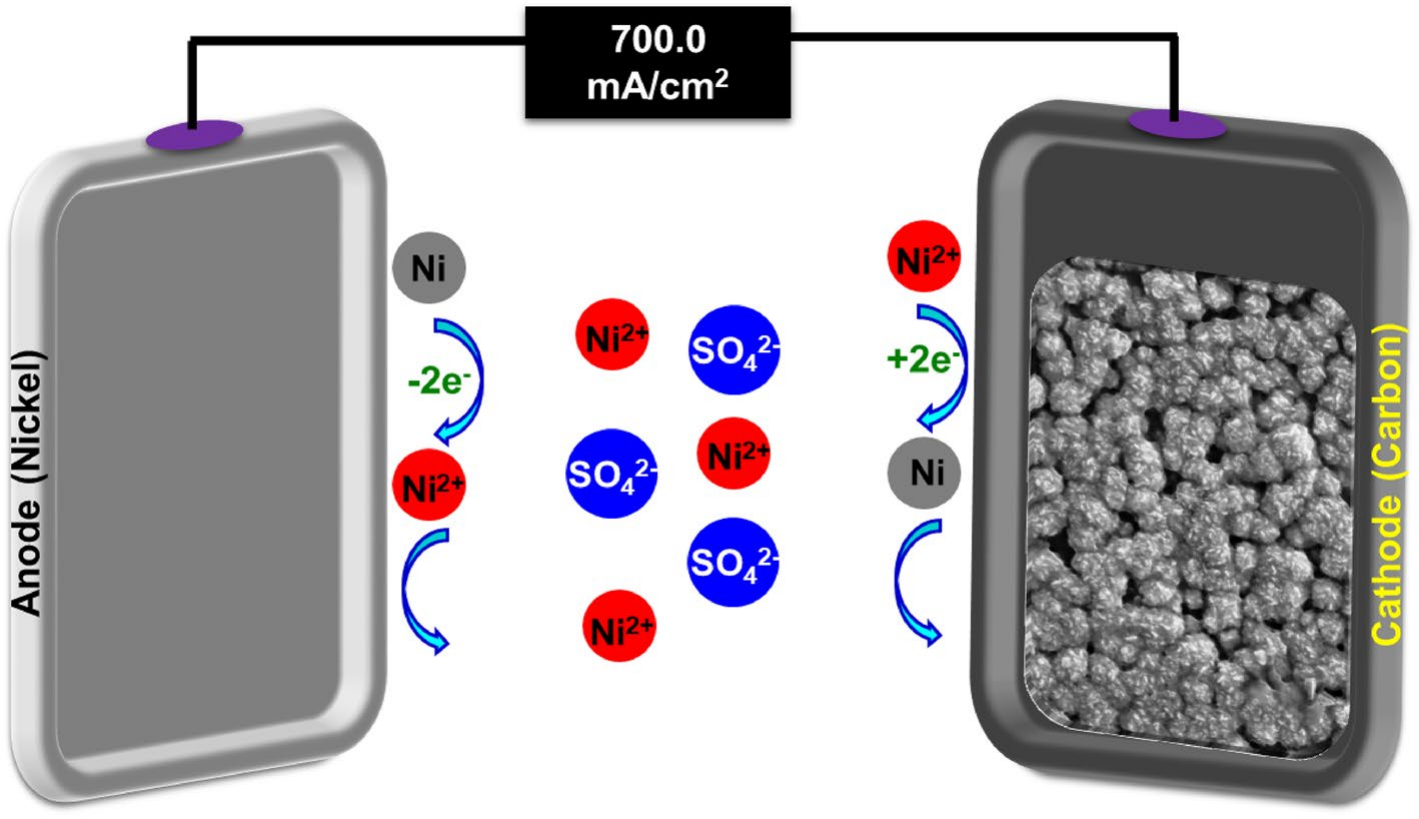
Electrodeposition of Nickel Film on the Graphite Plate.

**Figure 2:**
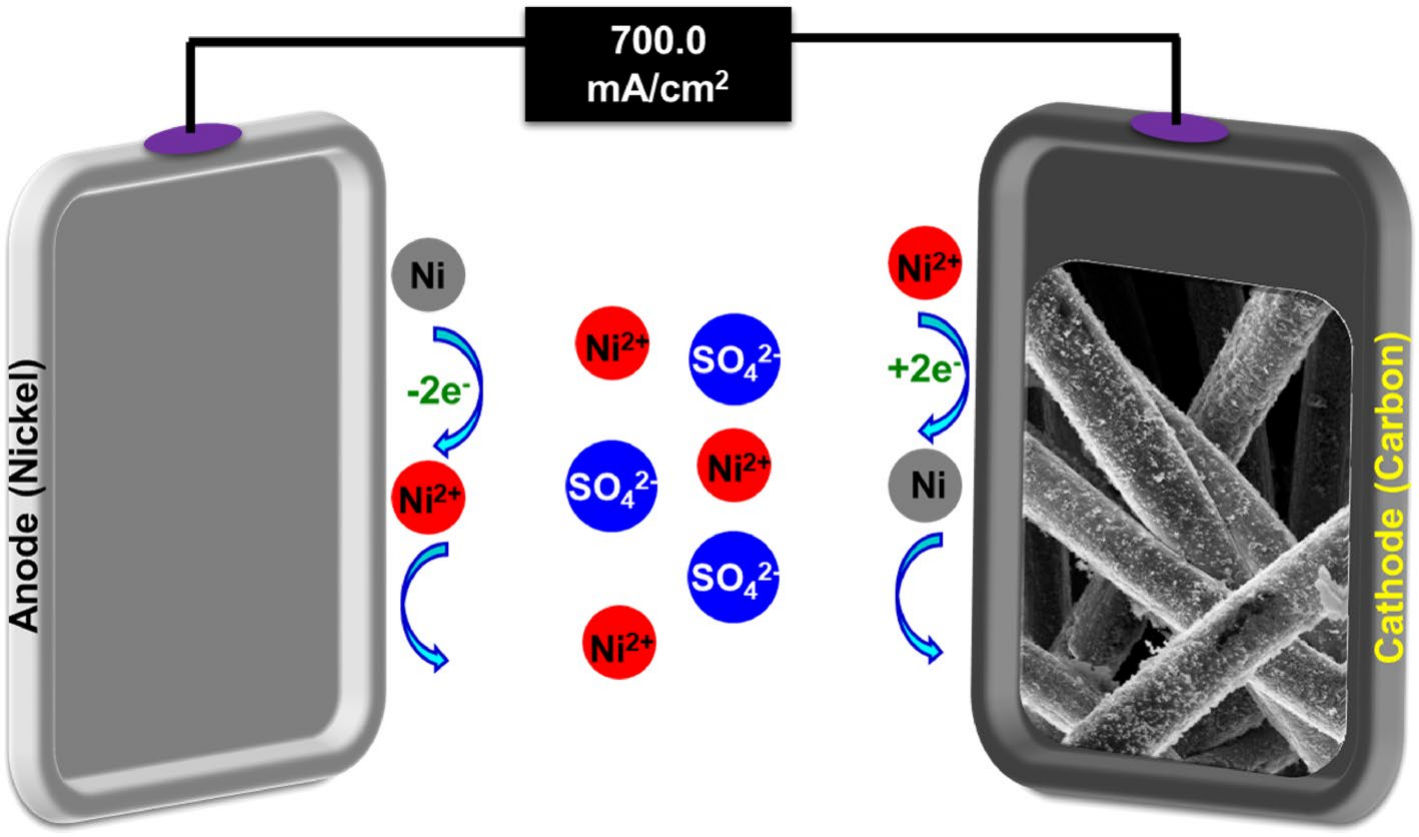
Electrodeposition of Nickel Film on the Carbon Felt.

The electroplating or cathodic electrodeposition method was employed to coat the surfaces of carbon felt and graphite electrodes with a nickel metallic thin film. Initially, the CF and GP electrodes were pretreated by soaking in acetone solution for 20 min, boiling in 0.1 M HCl for 15 min, and then washing with water to remove any oils and foreign particles from their surfaces^39^. The electroplating process was carried out in an undivided homemade reactor consisting of a metallic nickel plate as the anode, graphite plate and carbon felt as the cathode, and nickel sulfate (NiSO_4_·6H_2_O) as the supporting electrolyte in an aqueous solution of boric acid (H_3_BO_3_) at room temperature (Table 1). By applying a suitable current density for a specific period of time, the metallic anode begins to oxidize and releases the desired metallic cations (Ni^2+^) into the solution. Simultaneously, the dissolved metallic cations undergo cathodic electrodeposition onto the graphite plate and carbon felt electrodes. Finally, the nickel-coated graphite plate and carbon-felt electrodes are removed from the solution and rinsed with water (Figs. 1 and 2).

**Table 1:** The concentration of electrodeposition elements.

Chemical name	Formula	Concentration (g)	Metric (g/L)
Nickel sulfate	NiSO_4_·6H_2_O	8.90	179.70
Ammonium chloride	NH_4_Cl	1.50	24.70
Boric acid	H_3_BO_3_	1.50	29.96

### Microbial fuel cell set-up and operation

**Figure 3:**
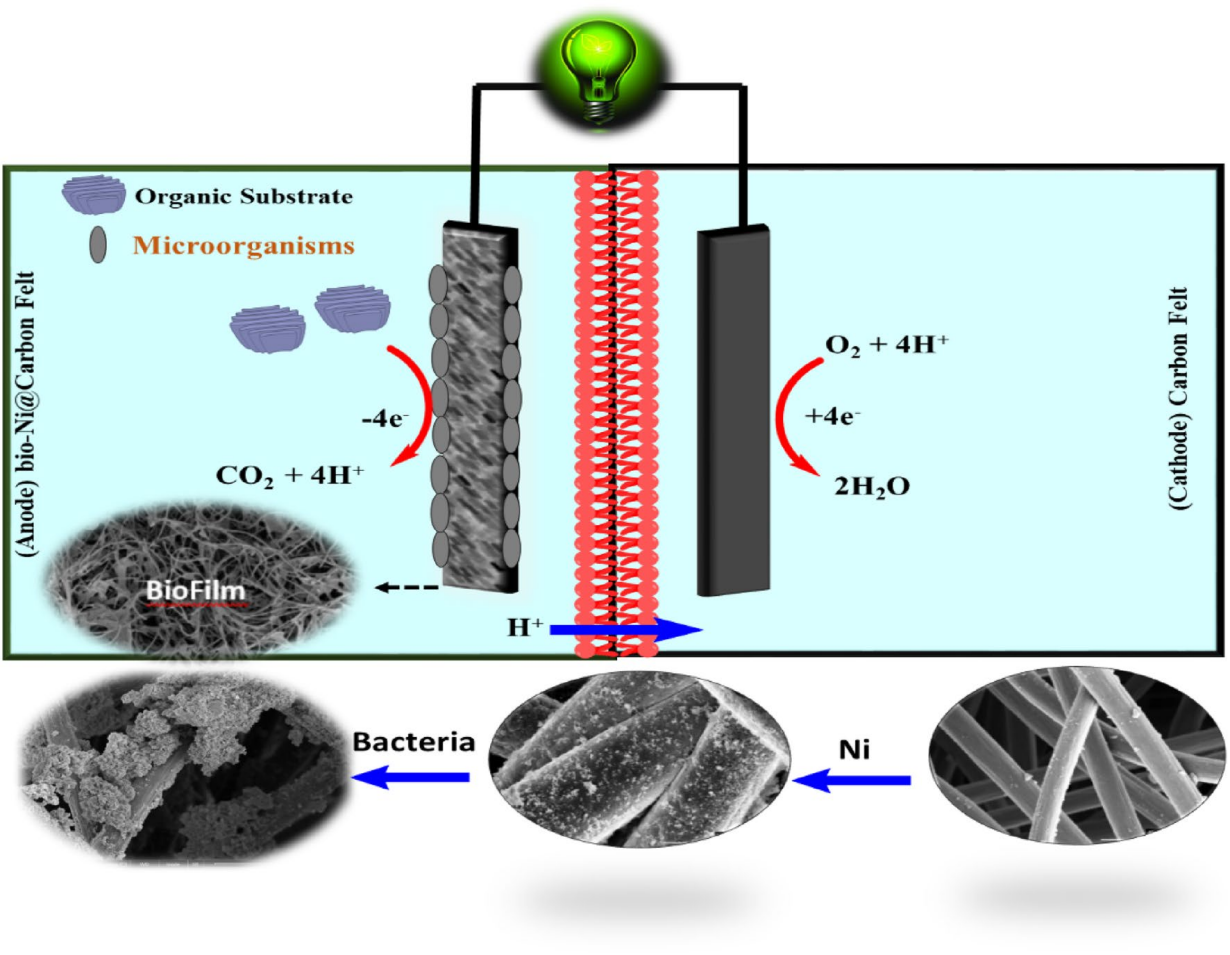
Schematic of the dual-chamber Microbial Fuel Cell (MFC) reactor.

This study was conducted using a dual-chambered plexiglass homemade (100 × 100 × 30 mm) Microbial Fuel Cell (DC-MFC) reactor with anodic and cathodic chambers, each with a volume of 350.0 mL^40,41^. The anodic and cathodic chambers were separated by a proton exchange membrane (Nafion 117, Sigma-Aldrich, USA) to facilitate the migration of H^+^ ions from the anodic to the cathodic chamber^42^. The Nafion membrane was pre-treated in five steps to remove any foreign contaminants and improve proton transportability. The membrane was washed sequentially with deionized water, H_2_O_2_ (3.0%), deionized water, 1.0 M HCl, and finally deionized water, with each step lasting for one hour at a temperature of 80 °C^43^. Carbon felt was used as the cathode in all reactors, while the anodes were made of bare graphite plate (GP), bare carbon felt (CF), nickel- coated carbon felt (Ni@CF), and nickel-coated graphite plate (Ni@GP), each with a surface area of 12.0 cm^2^ (40.0 mm * 30.0 mm * 3.0). The MFC reactor operated in batch mode at a constant temperature of 30 ± 1 °C and atmospheric pressure. Anaerobic sludge procured from the wastewater treatment plant in Urmia city was utilized as the source of requisite microorganisms for the anode chamber. To foster the growth and proliferation of these microorganisms and to prepare the inoculum, they were introduced into a culture medium (g/L) composed of 0.5 ammonium chloride, 5 glucose, 3 yeast extract, and 1 peptone. This mixture was subsequently incubated under anaerobic conditions for a duration of 24 h at a temperature of 30 °C. A v/v ratio of 10% of the prepared inoculum was introduced into the anode chamber^6^. Additionally, glucose at a concentration of 0.5 g per liter was employed as a carbon substrate and synthetic wastewater containing K_2_HPO_4_ (1.4), KH_2_PO_4_ (0.25), NH_4_Cl (0.31), MgSO_4_ (0.1), KCl (0.13), NaCl (0.1), and 1 mL/L of a mineral solution (g/L) encompassing FeCl_3_.6H_2_O (1.5), H_3_BO_3_ (0.15), CuSO_4_.5H_2_O (0.03), KI (0.03), MnCl_2_.4H2O(0.12), NaMoO_4_.2H_2_O (0.06), ZnSO_4_.H_2_O (0.12), and CoCl_2_.6H_2_O (0.15) was utilized in the anode chamber^39,44^. The anolyte was adjusted to a pH of 7.2 by phosphate buffer for microbial activity in the seed sludge^39^. Prior to the start of each run and the addition of inoculum, the anolyte was purged with N_2_ gas for 5 min to create an anaerobic environment and remove any oxygen^6^. In addition, in the cathode chamber, a buffer of 100 mM was employed, and dissolved oxygen was consistently supplied via the utilization of an air pump^45^ (Fig. 3).

A data logger device (DGHS-EL 2501, Danesh Gostar Hamgam Ba Sanat Company, Iran) for data recording was interfaced with a computer system. This system, equipped with MINITAB and MATLAB software, possesses the capability for online recording of open circuit voltage (OCV). Within the reactor setup, to capture OCV without the application of resistance, the generated voltage was recorded online at 30-min intervals. Upon establishing stable conditions and generating a constant voltage, the maximum power density and current density were ascertained by applying variable resistances ranging from 0.01 to 100.0 kΩ. Subsequently, the maximum power density (mW/m^2^) and current (mA/m^2^) were normalized in accordance with Eqs. (3) and (4), and voltage and power density curves were plotted against the current^46^.

3 $$\mathrm{I}=\frac{\mathrm{V}}{\mathrm{R}}$$

4 $$\mathrm{P}=\mathrm{IV}$$

where I, R, P, and V indicate the produced current, electrical resistance, power density, and cell voltage, respectively.

### Instrumental section

Field-emission scanning electron microscopy (FE-SEM; JEOL JSM-840A, Japan) was employed to investigate the surface morphology of the bio-anodes. The presence of nickel (Ni) on the bio-anodes was confirmed using energy-dispersive X-ray spectroscopy mapping (EDX; Bruker XFlash6L10) and elemental mapping. The crystal patterns of the deposited Ni thin film were analyzed using X-ray diffraction (XRD; Ultima IV, Rigaku). The voltage produced by the MFC was recorded using a data logger system (DGHS-EL2501, Danesh Gostar Hamgam Ba Sanat Company, Iran).

## Results and discussions

### Characterization of modified electrodes

**Figure 4:**
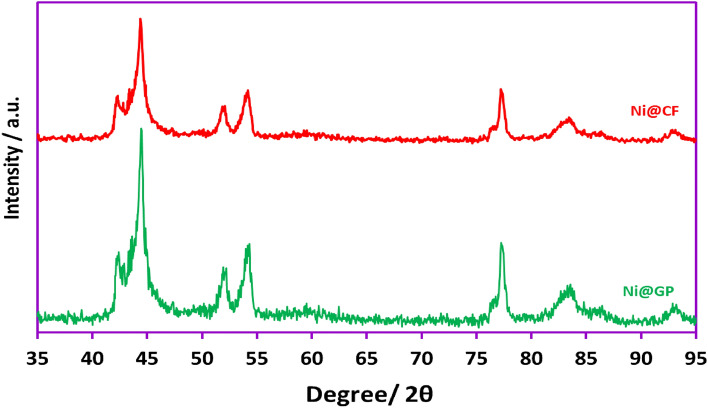
XRD patterns of the Ni@GP and Ni@CF electrodes.

In this study, the electroplating or cathodic electrodeposition method was utilized to coat graphite plate (GP) and carbon felt (CF) electrode surfaces with a nickel metallic thin film. The modification and various characteristics of the nickel-coated GP (Ni@GP) and nickel-coated CF (Ni@CF) electrodes were confirmed and characterized using X-ray diffraction (XRD), energy-dispersive X-ray spectroscopy (EDS), elemental mapping, and field-emission scanning electron microscopy (FE-SEM) techniques. The crystal structure of Ni@GP and Ni@CF was investigated using XRD (Fig. 4). The well-defined crystal structure of the coated nickel thin film was evidenced by the wide-angle XRD pattern, as reported in a previous publication^47^. The observed distinct peaks at 2Ɵ = 44.22°, 51.9° and 77.0° were attributed to the diffraction planes of [100], [40] and [20], respectively, confirming the synthesis of Ni nanoparticles on graphite plate and carbon felt based on the referenced JCPDS Cards Number of Ni-3600-r1.

**Figure 5:**
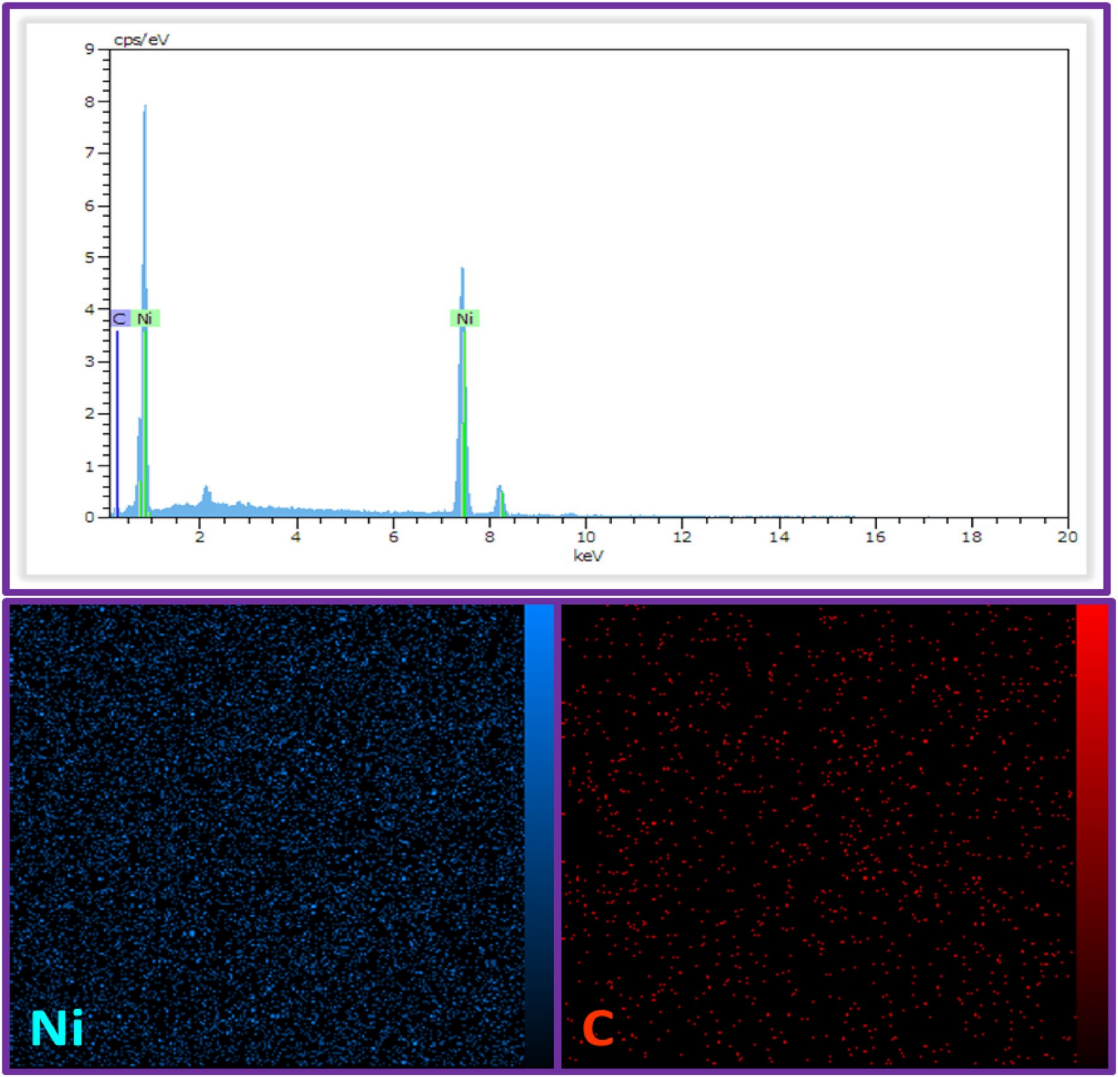
EDX spectrum and mapping element of the Ni@GP electrode.

**Figure 6:**
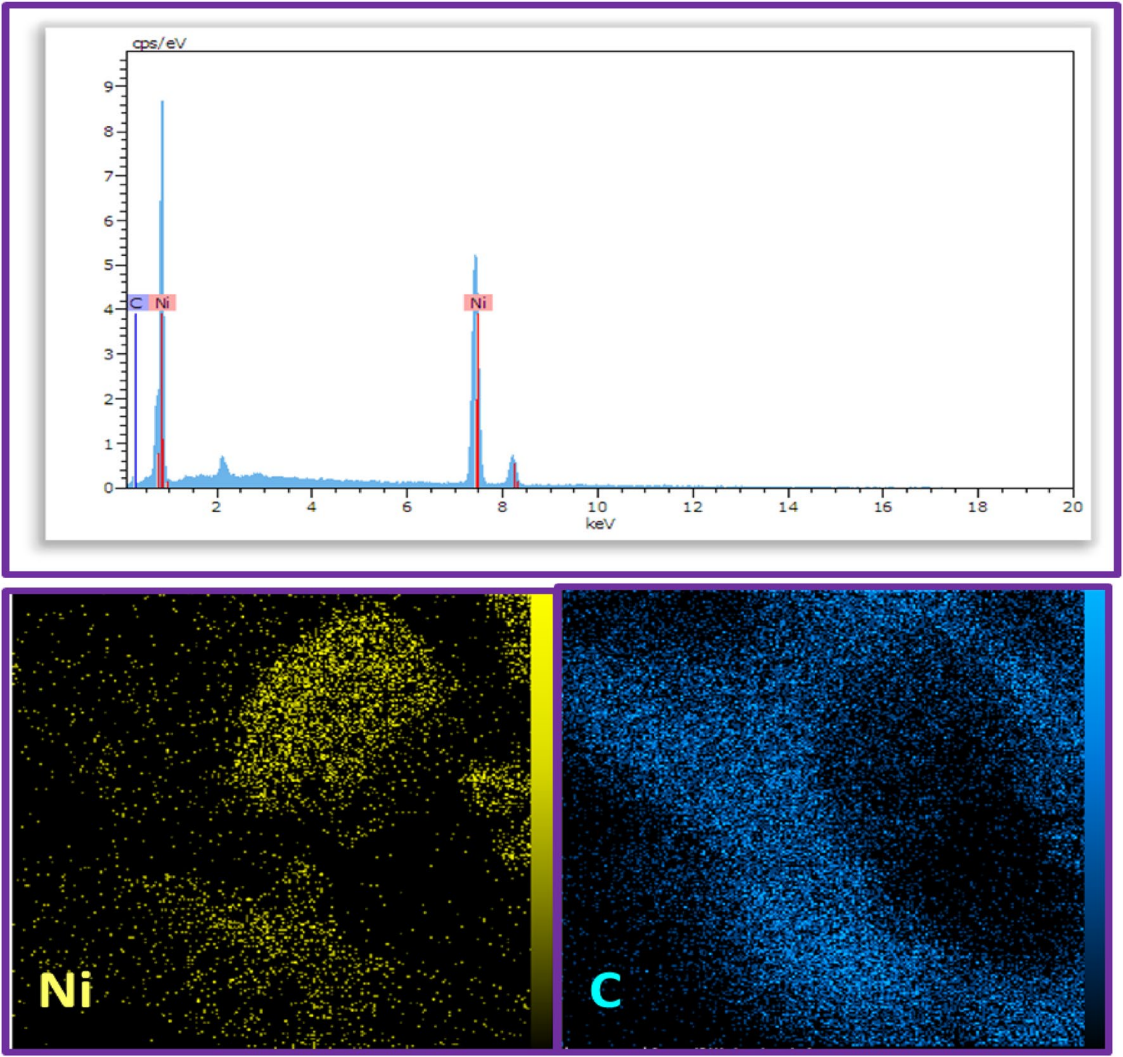
EDX spectrum and mapping element of the Ni@CF electrode.

Figures 5 and 6 depict the energy-dispersive X-ray (EDX) spectrum and elemental mapping images of the Ni@GP and Ni@CF electrode surfaces, respectively. The elemental analysis of the Ni@CF and Ni@GP electrodes reveals distinct peaks corresponding to nickel (deposited ions) and carbon (underlying substrate), confirming the accuracy of the electroplating procedure (Figs. 5-Up and 6-Up). Furthermore, the homogeneous and uniform distribution of nickel on the carbon plate and graphite felt is evidenced by the presence of nickel in the structure (Figs. 5-Down and 6-Down).

**Figure 7:**
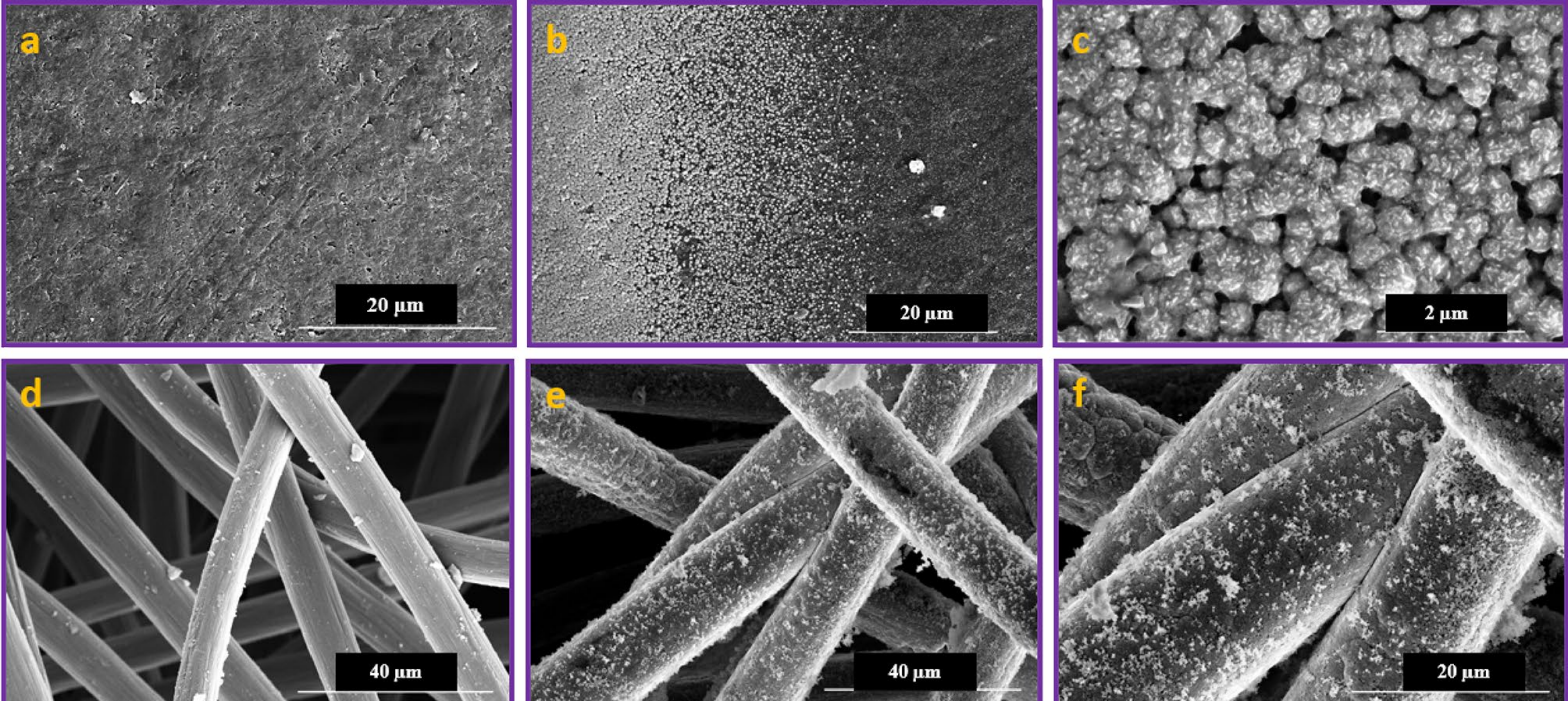
FE-SEM images of the (**a**–**c**) Ni@GP and (**d**–**f**) Ni@CF electrodes in different magnifications.

Figure 7 presents the field-emission scanning electron microscopy (FE-SEM) images of the bare and nickel-coated carbon felt (CF) and graphite plate (GP) electrodes. As can be seen from the large and close-up views of the recorded images, nickel nanoparticles have been continuously deposited onto the CF and GP electrode surfaces, in contrast to the bare electrodes. The observed morphological images reveal that the size and dimensions of the prepared and deposited nickel nanoparticles on the CF are smaller than those on the GP, due to the larger surface area of the stranded CF compared to the flat GP under constant applied current density for a fixed duration.

**Figure 8:**
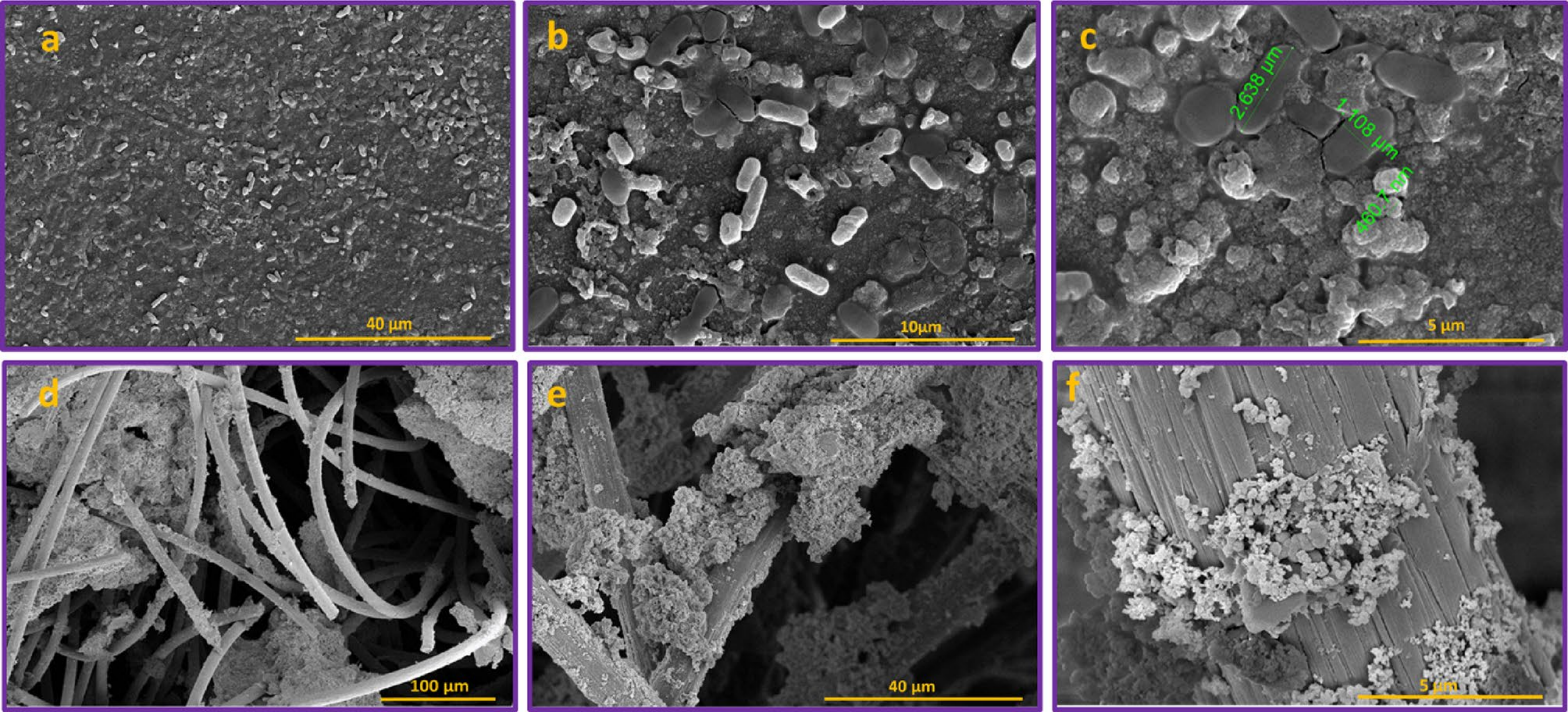
FE-SEM images of the (**a**–**c**) bio-Ni@GP and (**d**–**f**) bio-Ni@CF electrodes different magnifications.

In the bio-anode synthesis procedure, the prepared nickel-coated carbon felt (Ni@CF) and nickel-coated graphite plate (Ni@GP) electrodes in a microorganism solution for one month during several loading were immersed in a microorganism solution to facilitate the growth and stabilization of a biofilm on the Ni-coated electrodes, resulting in the fabrication of bio-Ni@CF and bio-Ni@GP electrodes. Figure 8 depicts the presence of microorganisms as a biofilm on the Ni-coated GP and CF electrodes. As mentioned before, To supply the microbial communities and form a biofilm layer, mixed active microorganisms from anaerobic sludge were used. Recognized species such as Klebsiella Pneumonia, Bacillus Subtilis, Shewanella, Enterobacter, Geobacter, Pseudomonas, Aeromonas. These electrogenic bacteria are capable of forming a biofilm layer and performing direct and mediated extracellular electron transfer(EET)^48^. A comparison of the images of the bio-Ni@CF and bio-Ni@GP electrodes, as shown in Fig. 8, reveals that, the characteristics of the anode surface have a significant impact on the colonization of exoelectrogenic bacteria. CF has a larger and more suitable surface area than GP for the penetration and attachment of microorganisms due to its high porosity^49^. GP may exhibit poor microbial attachment due to its uniform and smooth surface^50^. In addition the use of Nanomaterials as a catalyst with high biocompatibility can enhance electron transfer, reduce internal resistance, increase energy generation, and improve the efficiency of microbial fuel cells due to increased interaction between the electrode surface and microorganisms^51^. A similar study conducted by Zhong et al. found that modification of the CF surface affected the microbial community and increased the formation of biofilm, leading to an increase in voltage, current density, and power output^18^. Additionally, the results of a study by Ouis et al. confirmed that modification of the graphite electrode with conductive substances increased electron transfer and power density, which was attributed to the high adhesion of bacterial species on the anode and the formation of a more stable biofilm^52^.

**Figure 9:**
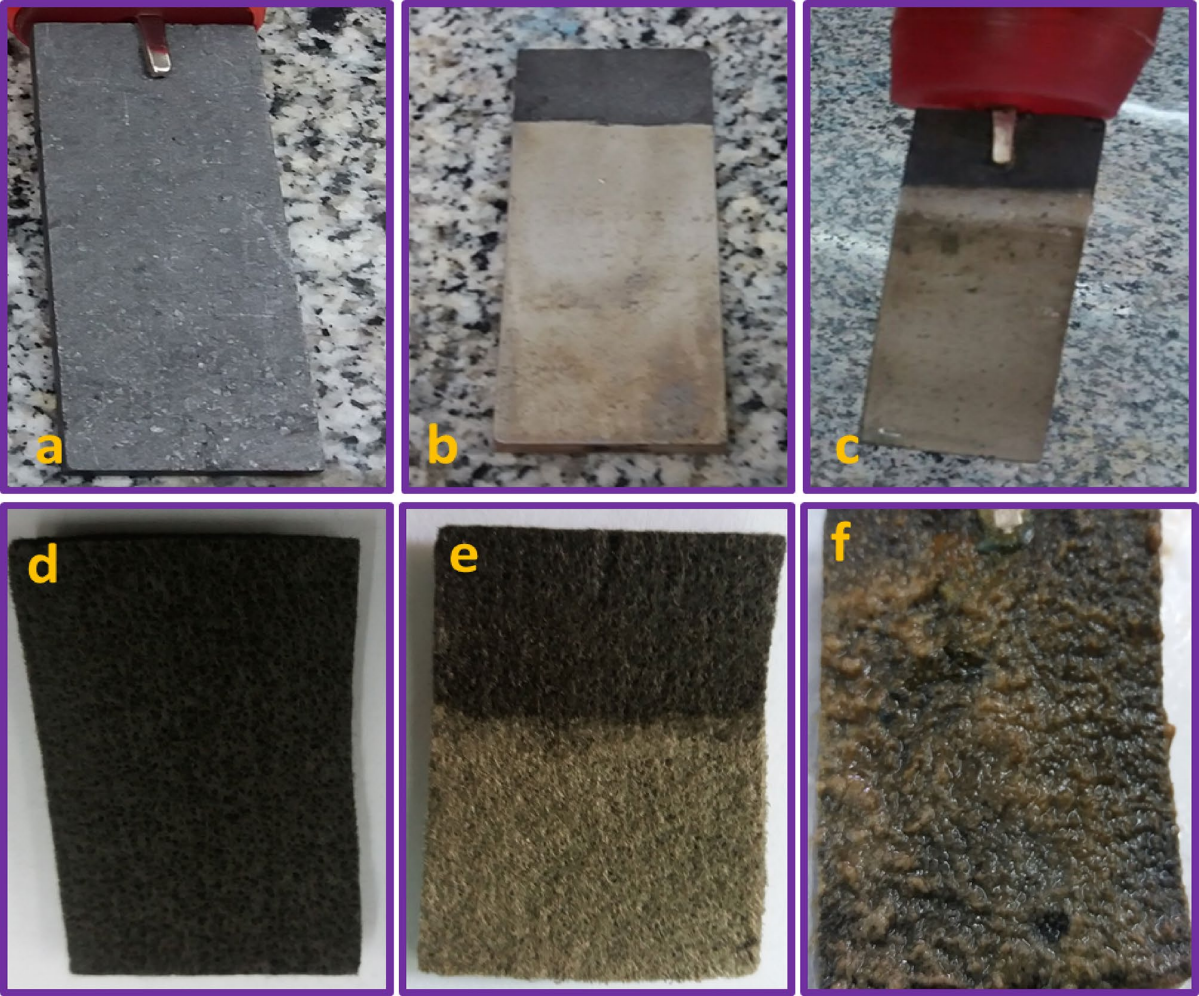
Real photographic images of the (**a**,**d**) bare GP and CF; (**b**,**e**) Ni@CF and Ni@CF; and (**c**,**f**) bio-Ni@CF and bio-Ni@CF electrodes.

Furthermore, Fig. 9a–f illustrates real photographic images of the bare GP and CF, Ni@GP and Ni@CF, bio-Ni@GP and bio-Ni@CF electrodes, respectively.

### Electrochemical performance of MFC

The open circuit voltage (OCV) of the MFC was automatically recorded using a data logger connected to a computer system systemwithout applying resistance every 30 min for 80 h. The voltage increased rapidly with each loading due to the addition of new substrate, then reached a steady state before decreasing due to substrate depletion and reloading. Monitoring the OCV data for four loadings of the MFC with different anodes indicated the suitable resistance and stability of the Ni film over time, as well as the development and growth of the biofilm.

**Figure 10:**
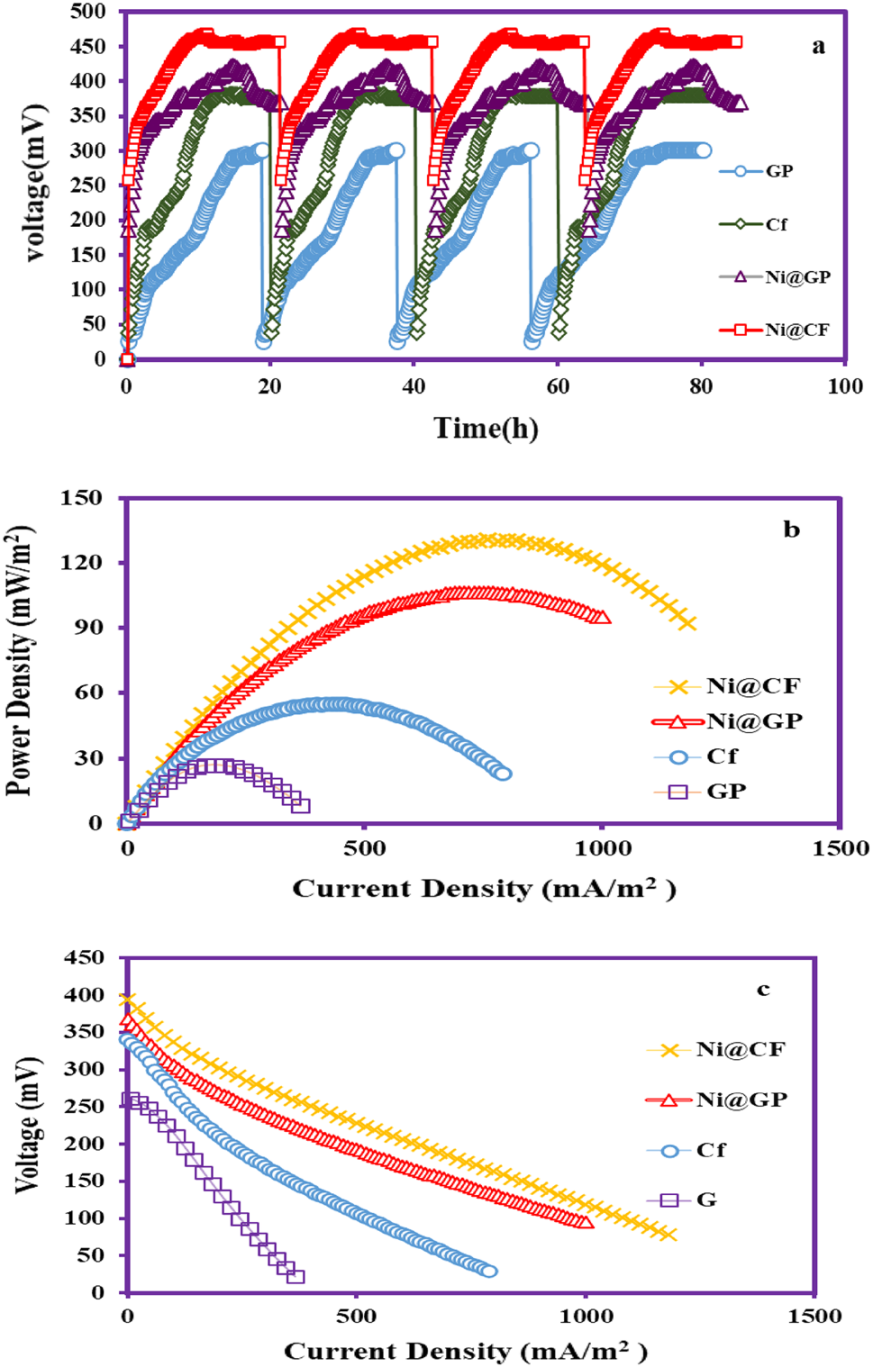
OCV (**a**) Power density and voltage as a function of current density (**b**,**c**).

According to the obtained OCV data, the highest average voltage output was observed using modified bio-Ni@CF (468.0 mV) and bio-Ni@GP (422.0 mV) electrodes, compared to bare CF(382.0 mV) and GP (301.0 mV) electrodes. Polarization and power density curves were obtained for different electrodes after several loading steps and biofilm formation, as well as ensuring a steady state of MFC conditions in terms of voltage generation by applying variable resistances (0.01–100.0 kΩ). As shown in Fig. 10b, the average maximum voltage output increased from 216.0 mV to 368.0, 382.0, and 394.0 mV for bio-Ni@CF, bio-Ni@GP, bio-CF, and bio-GP electrodes, respectively.

As shown in Fig. 10c, the type of electrode used had an effect on increasing power density output in the MFC system. The maximum power density and current density using the bio-Ni@CF electrode were 130.72 mW/m^2^ and 760.0 mA/m^2^, respectively. On the other hand, the maximum power density using bio-Ni@GP, bio-CF, and bio-GP reached 106.58, 55.04, and 27.01 mW/m^2^, respectively, which was 1.22, 2.37, and 4.83 times lower than that of the bio-Ni@CF electrode. Furthermore, the maximum current density for bio-Ni@GP, bio-CF, and bio-GP electrodes was 185.0, 430.0, and 730.0 mA/m^2^.

Therefore, as shown in Fig. 10, the bio-Ni@CF electrode with maximum voltage, power density, and current density was determined to be the optimal electrode and most suitable alternative material for improving MFC performance and electricity generation.

This study found that the efficiency of the modified carbon felt electrode was higher than that of the modified graphite electrode. Carbon felt has a porous structure due to its dispersed carbon fibers, as shown in the corresponding field-emission scanning electron microscopy (FE-SEM) images, which allows for greater attachment of electrogenic microorganisms to its surface and increased electron transfer rates compared to the smooth surface of graphite. A study by Rashidi et al. compared the performance of carbon felt and graphite electrodes in a two-chamber fuel cell and found that the maximum voltage, power density, and current density achieved using carbon felt electrodes were 1.38, 3.97, and 1.87 times higher, respectively, than those achieved using graphite electrodes.

The increase in power production observed with carbon felt was attributed to its highly active surface and structure compared to the smooth surface of graphite^45^.

Modifying the anodic electrode with nanomaterials to enhance bacterial growth on the anode and strengthen microbial attachment leads to improved performance and increased efficiency of microbial fuel cells (MFCs). As shown in Fig. 10, the bio-Ni@GP and bio-Ni@CF electrodes exhibit the highest energy generation efficiency compared to the bare GP and CF electrodes. In other words, nickel acts as a catalyst to increase conductivity and improve the electroactive properties of the electrodes. The investigation into the maximum power and current output using nickel foam as an anode, by Karthikeyan et al.^53^ also emphasized that the emproved of anode structural significantly effective in inhansing the generation power and current density. In another study Tahir et.al observed that the maximum power and current density in modified anodes significantly surpassed those of Carbon Felt (CF). In fact, the maximum power and current output using NiFe2O4-MXene@CF were 5.6 and 6.6 times higher than CF, respectively^22^. The power generated in the two mentioned studies showed a significant increase compared to our study, and this difference can be attributed to the fact that nickel particles were used in the present study, while in the mentioned studies, the synergistic effect of different compounds led to an increase Conductivity and more load transfer, also the use of different substrates in the anode chamber can be one of the factors influencing the production power. Yang et al. reported that the maximum power and current output obtained by the modified CF/Bio-Feox electrode were 71.64% and 59.21% respectively, higher than that of CF due to the high conductivity of metal oxide and high catalytic activity of the modified anode, leading to increased electron transfer, reduced internal resistance, and ultimately increased power output^54^. Similarly, Zhang et al. reported that the power density of the graphite/polyaniline-tourmaline electrode as a bio-cathode was 492.6% and 192.8% higher than that of unmodified cathodes and graphite/aniline cathode, respectively^55^. Table 2 shows the comparison of the present paper with previously reviews on the application of nanomaterial in MFCs.

**Table 2:** Comparing of the present paper with previously reviews on the application of nanomaterial in MFCs.

Anode	Cathode	Substra	Power density (mA/m^2^)	Current density (mA/m^2^)	Refs.
CF	CF	Glucose	247	153	22
NiFe_2_O_4_-MXene@CF			1385	1012	
NF	GP	Bad wine	8.2	25.8	53
NFPANI/TC/Chit			18.82 (W/m^3^)	54.9 (A/m^3^)	
CF/Bio-Feox	Pt/C	Glucose	797.0	226.1	54
GP	GP	Glucose	54	232	55
	Tourmaline/polyanilne modified graphite		266		
CF	CF	Glucose	55.04	430.0	This study
Ni@CF			130.72	760.0	

The results obtained in this study, as well as previous studies, have demonstrated that Ni can be used as a stable catalyst compatible with the conditions of microorganisms in the bio-anode. One of the factors affecting the stability of doped nanoparticles on the electrode is the doping method^19^. In previous reports, Ni was used as a catalyst in the cathode to modify activated carbon, which led to a reduction in electrode conductivity and electrical performance due to the use of polytetrafluoroethylene (PTFE)^56^. However, in the present study, the direct and in-situ electroplating or cathodic electrodeposition process was identified as an applicable method for the resistant and stable doping of nanoparticles on the electrode. As shown in Fig. 10a, the voltage output of four loadings using Ni@CF and Ni@GP electrodes did not change in the bio-anode. It should be noted that the results obtained in this study are consistent with those reported by Jia Liu et al. on the use of Ni, Fe, and Ni/Fe as cathode catalysts. Their study indicated that the use of Fe and Ni nanoparticles stimulated and increased bacterial growth on the cathode. However, over time, there was a reduction due to the destruction of the Fe catalyst. In contrast, Ni had the greatest effect compared to Fe and Ni/Fe on bacterial growth due to the relative stability of Ni nanoparticles compared to other catalysts^57^.

**Figure 11:**
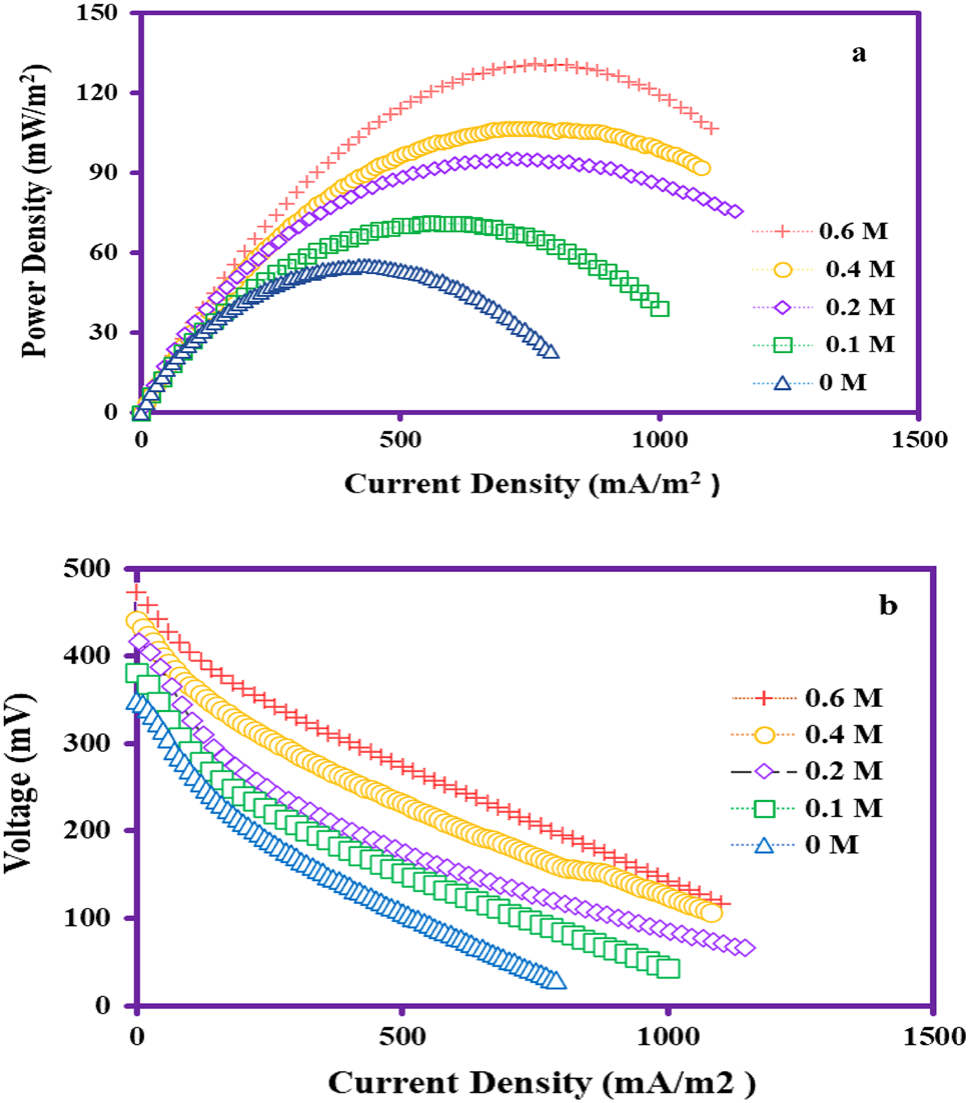
Power density and Polarization curves with various nickel catalyst concentrations.

Given that the concentration of the coated catalyst can be an important parameter for improving the performance of microbial fuel cells (MFCs), the maximum voltage, power density, and current density output at different concentrations of nickel (Ni) on the carbon felt electrode were investigated to determine the optimal electrode (Fig. 11). Therefore, optimizing the concentration of the catalyst is crucial for improving and modifying the surface of anode electrodes. According to Table 3, the maximum voltage, power density, and current density output were obtained in the presence of bare carbon felt and modified carbon felt at concentrations of 0.2, 0.4, and 0.6 mg/cm^2^ of Ni. The results shown in Fig. 11a,b indicate an increase in maximum voltage, power density, and current density with increasing concentration of the utilized catalyst in the anodic chamber. The lowest voltage, current density, and power density were attributed to the bare carbon felt electrode, while the highest voltage, current density, and power density were obtained for the modified carbon felt electrodes with a concentration of 0.6 mg/cm^2^, indicating increases of 25.97%, 43.42%, and 57.89%, respectively. Increasing the concentration of employed catalyst in the structure of the anodic electrode can be effective in increasing power density and improving the performance of MFCs due to the increase in surface area and high conductivity of nanomaterials^57^. However, the results of the present and previous studies have shown that using higher concentrations above threshold limits can have a negative impact on microbial growth and activity due to increased toxicity and reduced biocompatibility, leading to microbial inactivation. On the other hand, increasing the concentration of the catalyst beyond threshold limits may lead to saturation of the electrode surface and reduced attachment of microorganisms on the electrode^58^.

**Table 3:** Electrical performance of MFCs using different concentrations of nickel.

Anode material	Conc. of Ni (cm^2^/mg)	Voltage (mV)	Power density (mW/m^2^)	Current density (mA/m^2^)
CF	–	350.0	55.04	430.0
Ni@CF	0.1	381.5	71.12	560.0
Ni@CF	0.2	417.0	95.18	705.0
Ni@CF	0.4	440.0	106.58	730.0
Ni@CF	0.6	472.8	130.72	760.0

This survey demonstrated that a Ni-coated carbon felt electrode, produced using the electroplating method, can be utilized as a high-efficiency and stable bio-anode material for bio-electrogeneration and wastewater treatment processes. Our team’s research on the use of bio-Ni@CF electrodes in microbial fuel cell (MFC) systems for electrogeneration and removal of various pollutants from water environments is ongoing.

## Conclusion

The results of this study demonstrated that nickel, as a catalyst with high biocompatibility, increases conductivity and improves the electrical properties of carbon felt and graphite electrodes as bio-anodes, thereby enhancing the performance and efficiency of microbial fuel cells (MFCs). The maximum voltage, power density, and current density produced using carbon felt as an optimal electrode were significantly higher than those of other electrodes, at 468.0 mV, 130.72 mW/m^2^, and 760.0 mA/m^2^, respectively. However, increasing the concentration of the employed catalyst in the bio-anode led to a decrease in microbial growth due to increased toxicity and electrode surface saturation. As such, a concentration of 0.5 mg/cm^2^ of nickel was determined to be optimal. Additionally, the electrochemical deposition method can be used as a green, versatile, and applicable method for the resistant and stable deposition of nanoparticles on bio-anode electrodes. Finally, the fabrication of bio-Ni@CF modified electrodes using electroplating techniques can be employed as a novel, efficient, and versatile bio-anode in different MFC systems for increasing conductivity, improving electron transfer kinetics, promoting higher growth and stabilization of microorganisms, enhancing power density, and improving wastewater treatment.

## Data Availability

The authors declare that the all data generated or analyzed during this study are included in this published article.
